# Visualizing the dynamics of tuberculosis pathology using molecular imaging

**DOI:** 10.1172/JCI145107

**Published:** 2021-03-01

**Authors:** Alvaro A. Ordonez, Elizabeth W. Tucker, Carolyn J. Anderson, Claire L. Carter, Shashank Ganatra, Deepak Kaushal, Igor Kramnik, Philana L. Lin, Cressida A. Madigan, Susana Mendez, Jianghong Rao, Rada M. Savic, David M. Tobin, Gerhard Walzl, Robert J. Wilkinson, Karen A. Lacourciere, Laura E. Via, Sanjay K. Jain

**Affiliations:** 1 Center for Infection and Inflammation Imaging Research,; 2 Center for Tuberculosis Research,; 3 Department of Pediatrics, and; 4 Department of Anesthesiology and Critical Care Medicine, Johns Hopkins University School of Medicine, Baltimore, Maryland, USA.; 5 Department of Chemistry, University of Missouri, Columbia, Missouri, USA.; 6 Hackensack Meridian Health Center for Discovery and Innovation, Nutley, New Jersey, USA.; 7 Southwest National Primate Research Center, Texas Biomedical Research Institute, San Antonio, Texas, USA.; 8 Pulmonary Center, Department of Medicine, Boston University School of Medicine, Boston, Massachusets, USA.; 9 National Emerging Infectious Diseases Laboratories, Boston University, Boston, Massachusetts, USA.; 10 Children’s Hospital of Pittsburgh, University of Pittsburgh School of Medicine, Pittsburgh, Pennsylvania, USA.; 11 Department of Biological Sciences, UCSD, San Diego, La Jolla, California, USA.; 12 National Institute of Allergy and Infectious Diseases (NIAID), NIH, Rockville, Maryland, USA.; 13 Molecular Imaging Program at Stanford, Department of Radiology and Chemistry, Stanford University, Stanford, California, USA.; 14 Department of Bioengineering and Therapeutic Sciences, School of Pharmacy and Medicine, UCSF, San Francisco, California, USA.; 15 Department of Molecular Genetics and Microbiology, Duke University, Durham, North Carolina, USA.; 16 SAMRC Centre for Tuberculosis Research, DST/NRF Centre of Excellence for Biomedical Tuberculosis Research, Division of Molecular Biology and Human Genetics, Faculty of Medicine and Health Sciences, Department of Biomedical Sciences, Stellenbosch University, Cape Town, South Africa.; 17 Department of Infectious Diseases, Imperial College London, London, United Kingdom.; 18 Wellcome Centre for Infectious Diseases Research in Africa and Institute of Infectious Disease and Molecular Medicine, University of Cape Town, Cape Town, South Africa.; 19 The Francis Crick Institute, London, United Kingdom.; 20 Tuberculosis Research Section, Laboratory of Clinical Immunology and Microbiology, and Tuberculosis Imaging Program, Division of Intramural Research, NIAID, NIH, Bethesda, Maryland, USA.

## Abstract

Nearly 140 years after Robert Koch discovered *Mycobacterium tuberculosis*, tuberculosis (TB) remains a global threat and a deadly human pathogen. *M*. *tuberculosis* is notable for complex host-pathogen interactions that lead to poorly understood disease states ranging from latent infection to active disease. Additionally, multiple pathologies with a distinct local milieu (bacterial burden, antibiotic exposure, and host response) can coexist simultaneously within the same subject and change independently over time. Current tools cannot optimally measure these distinct pathologies or the spatiotemporal changes. Next-generation molecular imaging affords unparalleled opportunities to visualize infection by providing holistic, 3D spatial characterization and noninvasive, temporal monitoring within the same subject. This rapidly evolving technology could powerfully augment TB research by advancing fundamental knowledge and accelerating the development of novel diagnostics, biomarkers, and therapeutics.

## Introduction

*Mycobacterium tuberculosis*, the causative agent of tuberculosis (TB), remains a global public health emergency, as it is one of the top ten causes of death worldwide and a leading cause of death from a single infectious agent (1). New biomarkers and shorter treatments are therefore urgently needed to curb the TB epidemic (2). The alarming rise of multidrug-resistant (MDR) and extensively drug-resistant (XDR) TB (3, 4) poses additional challenges to TB treatment and signifies a need to not only expand the TB drug pipeline but also optimize the use of current and new TB drugs. Despite successes in the development of better diagnostic tools (5) and therapeutics (6), a lack of fundamental knowledge of TB pathogenesis and host-pathogen interaction dynamics hinders the development of innovative new tools, effective vaccines, and strategies for TB elimination (7).

### M.

**Figure 1:**
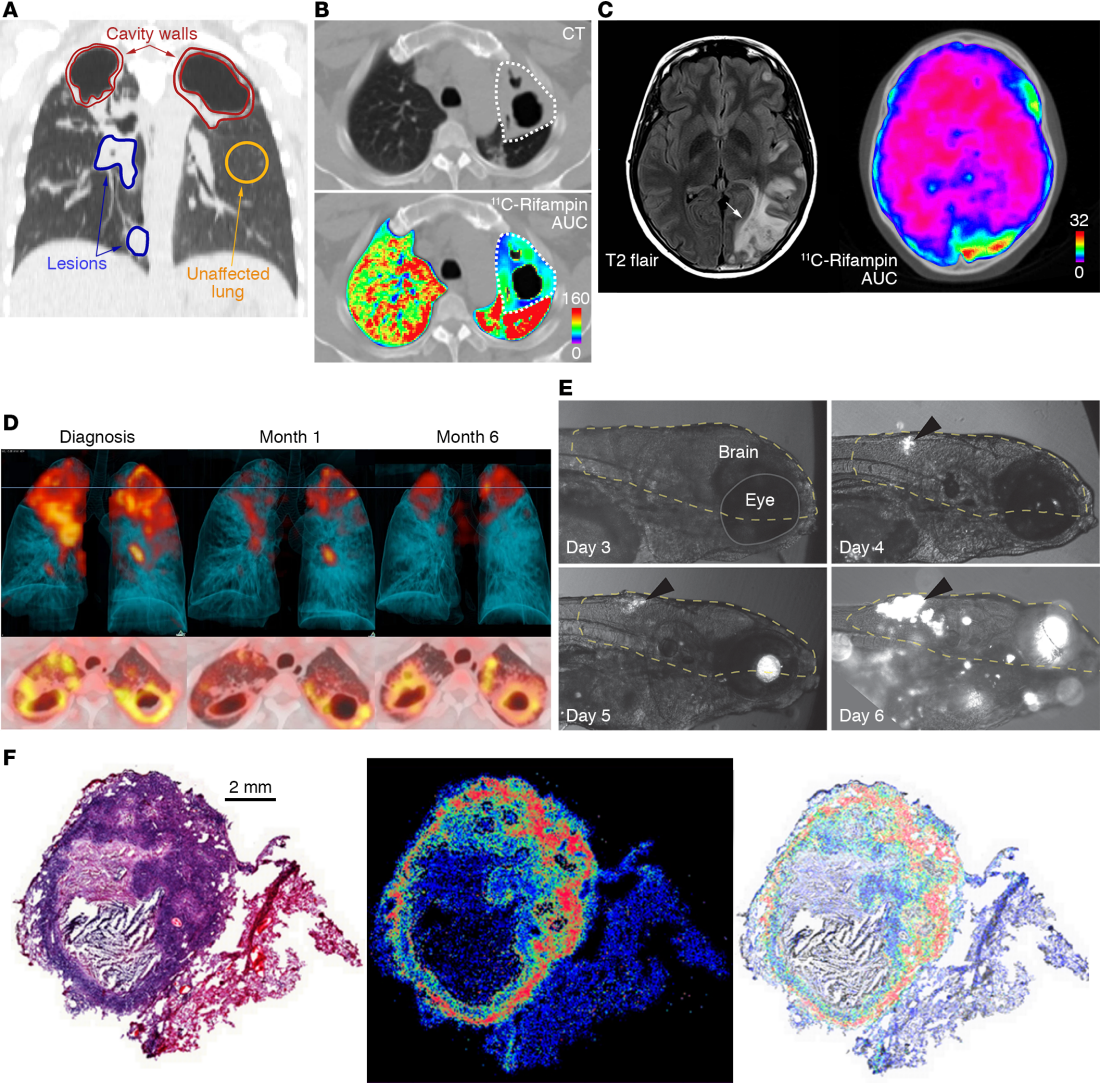
Spatial and temporal heterogeneity in TB lesions. (**A**) Coronal CT section from a TB patient with newly diagnosed cavitary TB demonstrating pathologically distinct TB lesions — granulomas (blue), pneumonia-like disease (blue), or cavities (red) — compared with unaffected lung (yellow). These different lesions demonstrate distinct pathological characteristics. (**B**) Radiolabeled ^11^C-rifampin PET/CT demonstrates spatially compartmentalized rifampin exposures in the pathologically distinct TB lesions within the same patient, with low cavity wall rifampin exposures. The ^11^C-rifampin AUC is shown as a heatmap overlay in the selected transverse section. **A** and **B** were adapted with permission from *Nature Medicine* (13). (**C**) MRI (T2 flair) demonstrates heterogeneous brain inflammation (arrow) in a patient with TB meningitis with the corresponding spatially heterogeneous ^11^C-rifampin AUC exposures. Adapted with permission from *Science Translational Medicine* (78). (**D**) Longitudinal ^18^F-FDG PET/CT in a cavitary TB patient over 6 months of standard treatment. Increased ^18^F-FDG uptake (compared with month 1) is noted at 6 months into treatment, coincident with treatment failure. **D** was adapted with permission from *EJNMMI Research* (108). (**E**) Fluorescence microscopy allows longitudinal imaging of the brain (dashed line) and eye (solid line) of a zebrafish larva infected i.v. with approximately 100 CFU of fluorescent *Mycobacterium marinum*:tdTomato. Infection began in the hindbrain ventricle (arrowheads). (**F**) Ex vivo H&E staining of a large rabbit necrotic TB granuloma (left), matrix-assisted laser desorption/ionization mass spectrometry imaging (MALDI-MSI) ion map of moxifloxacin in the same section (middle), and coregistration and overlay of moxifloxacin ion image with H&E staining (in grayscale) demonstrating accumulation in macrophage-rich regions (right). Scale bar: 2 mm. Images in **F** are courtesy of Drs. Landry Blanc and Véronique Dartois.

*tuberculosis* is notable for complex interactions with the host, leading to diseased states that range from subclinical infection to active disease and varied pathological lesions, such as necrotic lesions, cavitation, bronchiectasis, fibrosis, and pneumonia. These pathologies often coexist simultaneously in the same patient, each with distinct local milieu (bacterial burden, antimicrobial exposure, host response; refs. 8–13). Studies in animal models and TB patients have demonstrated that individual TB lesions within the same host are independent and asynchronous (9, 10, 12, 13). There is also spatial heterogeneity even within the TB lesion, with intralesional differences in bacterial burden and immune response (14–16). Unfortunately, conventional methods, which are primarily based on assays on clinical samples (e.g., sputum, blood, cerebrospinal fluid) or resected tissues, cannot optimally capture the heterogeneity or the temporal changes associated with disease progression or treatment (Figure 1). For instance, clinical samples may not correlate well with the lesion biology at infection sites where the pathogen resides. Moreover, because of the difficulties of obtaining direct tissue and the risk of sampling bias due to lesion heterogeneity, data on human lesion biology remain limited. Essentially, TB is a 21st century global health threat; however, the field continues to be reliant on several diagnostic and research tools developed more than 100 years ago. In contrast to conventional methods that use preserved tissue samples (e.g., histology) to obtain molecular information, molecular imaging approaches focus on imaging molecules of interest within living subjects. Therefore, in this Review, we will focus primarily on imaging of intact living subjects (Table 1), while also highlighting the complementary contributions that other ex vivo imaging technologies, such as matrix-assisted laser desorption/ionization mass spectrometry imaging (MALDI-MSI) and autoradiography, have made to the TB field.

Tomographic imaging can evaluate disease processes deep within the body, noninvasively and relatively rapidly, by providing 3D information about the disease and local biology throughout the body, and is less prone to sampling errors (Table 2). CT provides anatomic information and is used extensively in the clinical management of several diseases, including TB. Chest CT is generally considered superior to chest radiography for identifying features consistent with TB, especially in children (17). Longitudinal CT has been used to monitor the dynamics of cavitary formation in animal models (18, 19) as well as TB treatment response in animals and humans (4, 8, 20–23). MRI provides high-resolution and -contrast anatomic imaging and can detect tissue necrosis (an important pathological feature of TB) with high sensitivity (24). Additionally, MRI has advanced capabilities, such as dynamic contrast-enhanced imaging, chemical exchange saturation transfer (CEST) contrast, and MR spectroscopy (MRS), that can detect physiological or metabolic changes without the need of exogenous agents. In animal models, these novel MRI capabilities were able to differentiate sterile inflammation or oncological processes from bacterial infections (25, 26).

Clinically available whole-body molecular imaging modalities also include nuclear medicine tools such as PET or single-photon emission computed tomography (SPECT), which are based on the detection of the energy produced by radioactive compounds administered in micromolar quantities to a patient or subject. These tools can provide unparalleled opportunities for visualizing infections, especially as molecular and metabolic alterations occur earlier than structural changes. The possibility of radiolabeling a very large number of compounds that can then be detected with PET/SPECT leads to a wide range of applications in clinical and preclinical research. These compounds can be synthesized to target specific biochemical processes that can then be detected and quantified (e.g., glucose metabolism with 2-[^18^F]fluoro-2-deoxy-d-glucose [^18^F-FDG] PET). PET- and SPECT-based technologies have augmented early diagnosis, monitoring, and investigation of various diseases (27). For example, ^68^Ga-DOTATATE PET can detect early neuroendocrine tumors with higher accuracy than conventional imaging modalities (28). Similarly, the implementation of prostate-specific membrane antigen–targeted (PSMA-targeted) PET agents has significantly improved the management of prostate cancer (29). While these tools are integral in the management of patients with cancer, molecular imaging is not widely used for infectious disease but has similar potential (30). PET and SPECT, combined with anatomic imaging (CT, MRI), allow noninvasive detection of dynamic biochemical changes in TB disease without altering the system. This allows the possibility of repeated studies in the same subject, providing longitudinal measurements in the same patient, representing a fundamental advantage over traditional tools. These data can then be used to inform mathematical models of disease progression, which represents a major advance for the field that has primarily relied on snapshots to understand TB. Spatial information enables therapeutic monitoring in patients with deep-seated infections for whom clinical samples (e.g., blood) may be noncorrelative to disease severity and biopsy may be risky or impractical.

Optical imaging provides high-resolution (e.g., single-cell resolution) live imaging in small animal models and has provided valuable insights into biological processes (e.g., TB granuloma formation). Ex vivo techniques can also provide detailed visualization of tissues, and MALDI-MSI is being increasingly utilized in TB research to spatially localize endogenous and exogenous molecules within tissue sections (31). MALDI-MSI is label-free and can localize ionized molecules (e.g., drugs, metabolites, lipids, proteins) and overlay them onto histologically stained sections to enable spatial distribution of each ion of interest with cellular and subcellular resolution (31, 32). MALDI-MSI can also be applied to archived tissue blocks dating back decades (33).

## Pathogen-specific molecular imaging

^18^F-FDG PET has been extensively used in TB patients and animal models to monitor and characterize disease (8, 20, 34, 35). Immune cells increase the use of glucose as an energy source during metabolic bursts associated with inflammatory responses due to infection, and this change in glucose utilization can be visualized with ^18^F-FDG PET with high sensitivity (36). ^18^F-FDG PET/CT has been successfully used to assess TB pathogenesis, bacterial dissemination, and disease progression in animal models that mimic different stages of pulmonary TB disease (37, 38). However, as an analog of glucose, ^18^F-FDG is unable to differentiate among oncological, inflammatory, and infectious processes. Therefore, pathogen-specific imaging agents are being developed to specifically detect *M*. *tuberculosis* complex bacteria. Radio-analogs (e.g., *para*-aminobenzoic acid [ref. 39] and trehalose [ref. 40]) that target specific metabolic pathways present in bacteria but absent in mammalian cells provide opportunities for whole-body imaging to detect *M*. *tuberculosis* (ref. 30; see Table 3 for a summary of molecular imaging agents that have been used for TB). Similarly, enzyme-activated substrate probes, such as those activated by mycobacterial hydrolases and proteases, can also serve as targets to develop pathogen-specific imaging agents (41). The ability to detect and quantify the bacterial burden using *M*. *tuberculosis*–specific imaging agents with high sensitivity has potential not only to improve diagnostic accuracy but also to allow the development of tools to noninvasively monitor treatment response and prognosticate disease. It should be noted that all whole-body pathogen-specific imaging approaches for *M*. *tuberculosis* are in early development. Therefore, detailed preclinical validation and subsequent human studies are required before clinical application.

Optical imaging is a complementary approach to PET/SPECT but is limited by the absorption of light within deep tissues (42), and therefore can be applied primarily to small animal research. For example, specific hydrolysis of a novel fluorescence reporter enzyme substrate by β-lactamase (BlaC), which is naturally expressed by *M*. *tuberculosis*, was used to image *M*. *tuberculosis* in situ in a mouse model with high sensitivity (43). Membrane-localized mycobacterial enzymes like BlaC, mycolyltransesterase, decaprenyl-phosphoryl-ribose 2′-epimerase (DprE1), and trehalose have been targeted to develop fluorogenic probes that can rapidly detect *M*. *tuberculosis* in sputum samples (44–46) and within macrophages (47). These enzyme-dependent probes can be used for time-lapse and fixed-cell imaging of mycobacteria in microfluidic devices to visualize single-cell biology (48). Finally, fluorescent reporters have been combined with bronchoscopy for specific detection of bacteria in distal human airways and alveoli (49).

## Biomarkers for disease stratification and outcomes

Disease heterogeneity has been a major barrier to clinical studies and patient care, as TB patients with different disease states are grouped together to receive the same treatment. For instance, since the 1970s and 1980s, it has been known that 80%–85% of patients with uncomplicated, drug-susceptible pulmonary TB may be successfully cured after 4 months of therapy (50). However, the current standard of care still requires a 6-month treatment regimen to avoid relapse, which occurs in a small subset of patients whose characteristics are largely unknown and who are therefore not readily identifiable. Although the 2-month sputum culture status is widely used to assess treatment efficacy in clinical trials and for patient care (51), its correlation with the risk of relapse after treatment completion has been disappointing (52), presumably because sputum bacterial burden only represents lesions in free communication with the airway and thus is not reflective of the total pulmonary disease burden. More recently, promising whole-blood transcriptomic signatures have been identified that correlate with the radiological extent of disease (53) and severity of lung inflammation (54), distinguish patients with active TB from latent infection (55), and potentially identify patients at risk for treatment failure (56). In addition to these advances, imaging techniques could serve as important complementary approaches to noninvasively characterize the diseased states (57).

In a small study in adults with MDR-TB utilizing ^18^F-FDG PET as a metabolic marker and CT to assess the radiological extent of disease, quantitative changes in computed abnormal volumes on CT or ^18^F-FDG uptake at 2 months into treatment were predictive of long-term outcomes in these patients (20). ^18^F-FDG PET/CT has also been used to accurately identify TB reactivation risk in animal models and human subjects (8, 23, 58). A prospective, multicenter, randomized phase IIb clinical trial is under way to evaluate whether baseline stratification of disease burden quantified by ^18^F-FDG PET/CT and changes in ^18^F-FDG PET/CT parameters at 1 month after treatment initiation can identify pulmonary TB patients who can be cured with 4 months of standard treatment (59). Although ^18^F-FDG PET is currently under investigation, more specific imaging biomarkers, such as ^124^I-DPA-713 PET (60, 61), and pathogen-specific molecular imaging approaches (39) could also be used to aid patient stratification, monitor response to treatment, and accelerate the development of novel therapeutics.

## Antimicrobial biodistribution

Effective treatment of infections depends on achieving adequate antimicrobial concentrations at infection sites, where the pathogen resides (62). However, because of the difficulties of direct tissue sampling, drug pharmacokinetic (PK) and pharmacodynamic (PD) studies have relied on sequential sampling of blood and rarely other fluids (i.e., cerebrospinal fluid or bronchoalveolar lavage) to measure drug levels. TB creates heterogeneous pathology with multiple disease states occurring simultaneously, with potentially different coexisting microenvironments. Inadequate antimicrobial levels at the site of infection are one important contributor to treatment failure and emergence of MDR and XDR strains (15). However, antimicrobial concentrations account for the majority of variance in TB treatment outcomes (failure, relapse, death), which can be abrogated by higher drug levels (63).

Ex vivo techniques such as MALDI-MSI can provide high-resolution 2D ion maps of molecules (e.g., lipids, proteins, etc.) and their metabolites (64). This technology has been used for nearly a decade to assess drug biodistribution in animal models of TB providing valuable data (65). For instance, Prideaux et al. used MALDI-MSI to demonstrate limited penetration of moxifloxacin into necrotic caseum versus higher levels in the surrounding cellular regions (66). Similarly, MALDI-MSI has confirmed that isoniazid rapidly and homogenously penetrates TB lesions with rapid clearance (67) and identified multiple patterns of drug distribution in lesions (68–71). A major advantage of MALDI-MSI is that multiple molecules, such as drugs and lipids, can be simultaneously detected from the same tissue (72). While extensive MALDI-MSI data are available from animal models, MALDI-MSI relies on tissue resection, which is only performed in patients when it is clinically indicated for disease management. Thus far, intralesional drug levels have been measured only in patients with refractory disease undergoing surgical resection for clinical reasons (66) and thus may not be representative of the vast majority of TB patients (73). Similarly, the acquisition of longitudinal measurements in the same subject is also challenging.

Whereas MALDI-MSI provides high-resolution drug distribution of resected tissue, PET imaging enables dynamic, longitudinal assessment of drug PK, albeit at a lower resolution. Several key and newer TB drugs, including rifampin, pyrazinamide, isoniazid, linezolid, and bedaquiline, have been radiolabeled to provide noninvasive PK data with PET imaging (Table 3 and refs. 13 and 74–77). PET imaging can directly visualize multiple compartments simultaneously and monitor changes over time, thereby reducing sampling bias. Coregistration of the PET signal with CT or MRI provides anatomic information and localization of the PET signal with disease pathology. By noninvasive repeat acquisitions, PET can also provide AUC measurements (the critical PD parameter for several TB drugs) or detect changes in antimicrobial distribution during treatment or disease progression (13). For instance, longitudinal PET imaging in a rabbit model of TB meningitis demonstrated a significant decrease in ^11^C-rifampin brain penetration as early as 2 weeks into treatment (78). Ordonez et al. demonstrated lower penetration of ^11^C-rifampin in cavitary walls, which are associated with a high bacterial burden, compared with other types of TB lesions as well as independent temporal evolution of different TB lesions in the same patient during treatment (13). Ex vivo autoradiography can also be used with other staining techniques, such as immunofluorescence, in animals after PET to provide high-resolution intralesional biodistribution similar to MALDI-MSI (74). Multimodality imaging can also provide valuable information on the interaction of the host response and drug penetration. However, PET imaging is limited by the radiological half-life, which can range from minutes to days, of the isotope used to radiolabel the drug and is unable to distinguish between the radiolabeled drug and its metabolites.

CT characteristics, such as presence or absence of cavitation, have also been used to stratify patients into easy-to-treat and hard-to-treat phenotypes in patient-level pooled analysis of shortened treatment regimens, and incorporation of additional molecular imaging PK data could provide imaging biomarker information (79–81). Together, the integration of data from molecular imaging and ex vivo techniques could build more accurate and sophisticated PK/PD models to optimize antibiotic treatments and improve outcomes in TB patients (82).

## Understanding host-pathogen interactions

Imaging-specific host responses could provide important insights into TB pathogenesis and have potential to serve as biomarkers to predict treatment response and accelerate therapeutic development. Importantly, disease outcomes in certain forms of TB, such as TB meningitis, may be more strongly associated with changes in intracerebral inflammation than with bacterial killing, as immunoinflammatory damage is a critical pathological process in this disease (83). Molecular imaging tools could allow noninvasive readouts of neuroinflammation in animal models as well as in human studies (60, 83).

Radiolabeled iodo-DPA-713, a synthetic ligand for the translocator protein (TSPO), is a specific imaging biomarker for microglia/macrophage-associated inflammation and was predictive of treatment efficacy and relapse in an *M*. *tuberculosis* murine pulmonary infection model (61, 84). In this model, iodo-DPA-713 imaging was found to be superior to ^18^F-FDG PET as a marker for treatment response, and an early increase in iodo-DPA-713 activity, but not ^18^F-FDG, correlated significantly with the bacterial burden at relapse (61). Additionally, ^124^I-DPA-713 PET imaging in a rabbit model of TB meningitis showed tracer colocalization with tuberculomas, and postmortem immunohistochemical staining confirmed microglial activation (83). Biodistribution and dosimetry studies demonstrated that ^124^I-DPA-713 PET was safe and well tolerated in humans with low pulmonary background signal (60). Therefore, ^124^I-DPA-713 PET is an example of a molecular imaging approach that could be translated to the clinic to monitor TB-associated microglia/macrophage inflammation.

Other PET ligands targeting various immune cells and biological processes (e.g., apoptosis and hypoxia) are also in development. cFLFLF, which targets formyl peptide receptor 1 (FPR1) expressed in neutrophils and some monocytes in peripheral blood, has been evaluated in animal models of TB (85). Similarly, ^64^Cu-LLP2A, a high-affinity peptidomimetic ligand for very late antigen-4 (VLA-4; also known as α_4_β_1_ integrin), has also been evaluated in *M*. *tuberculosis*–infected macaques, where VLA-4 was found to be expressed primarily by macrophages and T cells, and to a lesser extent by neutrophils and B cells (86). Molecular imaging can also be used to understand TB pathogenesis and host-directed pharmacological interventions. For example, *M*. *tuberculosis* proliferates in macrophages during the early phase of infection and induces antiapoptotic proteins, leading to necrosis of the infected cells and subsequent tissue destruction (87, 88). In addition to tissue destruction, necrosis also reduces antibiotic penetration and access to immune cells in the infected sites. Therefore, proapoptotic drugs could be used as host-directed therapies that could be noninvasively monitored using caspase-3/7–specific PET agents like ^18^F-ICMT-11 and ^18^F-C-SNAT (89, 90). Similarly, hypoxia-targeting imaging agents have been used in TB to evaluate tissue damage and response to treatment (91, 92).

Although the use of molecular imaging in vaccine development is still in its infancy, it holds promise to elucidate mechanistic information, visualize spatiotemporal dynamics of immune responses, and expedite vaccine development. ^18^F-FDG PET/CT can be used to noninvasively monitor disease progression in animal models after immunization (93). Similarly, molecular imaging with probes that target specific immune cells can provide valuable information to elucidate the spatiotemporal kinetics of immune responses elicited by a vaccine or subsequent challenge by the pathogen (94, 95). Immune cell–specific imaging could provide information on when and where optimal immune responses develop. Molecular imaging could also be used to monitor “vaccine-take,” a successful immune response to the vaccine, in deep-seated sites, such as pulmonary lymph nodes, in animal models as well as human subjects (96). Moreover, this approach could be used to assess vaccine effect on key immune types and inform optimum vaccine dose and administration route. Finally, molecular imaging can also provide data to identify biomarkers of disease characteristics that could be used to select participants for clinical trials or stratify individuals at enrollment of therapeutic or vaccine trials to control for disease heterogeneity.

Optical imaging can provide complementary data when combined with other methods. For example, while CD4^+^ T cells must have direct contact with *M*. *tuberculosis*–infected cells to provide immunological protection, the mechanisms that restrict this process remain poorly understood (97, 98). However, fluorescent microscopy of engineered mycobacterial strains and cells, including fluorescent reporter T cells specific for activation by antigen, in animal models can overcome these limitations to better evaluate these processes. For instance, the use of bacterial reporter strains allows imaging of the bacteria within thick lung tissue via fluorescence confocal microscopy, and thus allows evaluation of host-pathogen interactions at a single-bacterium level while retaining the intact lung architecture (99). Techniques such as fluorescence-lifetime imaging microscopy (FLIM) allow the simultaneous use of multiple fluorophores in the same tissue to evaluate different pathological processes. Similarly, *Mycobacterium marinum* and *Mycobacterium leprae* can be used in the zebrafish model (100, 101), which, owing to the optical transparency of the larvae, allows direct visualization of granuloma formation. Recently, mature, fully organized zebrafish granulomas have been microdissected and maintained in 3D in vitro cultures, allowing direct visualization of the granuloma and screening of chemical libraries for potential treatments (102, 103). Multiphoton intravital microscopy (MP-IVM) can also provide quantitative live cell imaging with high contrast, specificity, and resolution. Recent developments have allowed imaging of 1–2 mm of lung tissue surface of live animals using an intercostal window (104) that could allow longitudinal tracking of granulomas located on the lung surface in live *M*. *tuberculosis*–infected mice (105).

Finally, ex vivo techniques such as MALDI-MSI have been used to interrogate inflammatory signaling lipids within TB lesions, which have been shown to be spatially organized within the developing granuloma (12). Elucidation of this information is critical to understanding host-pathogen interactions during infection, and spatial organization of TB lesions is currently being investigated. Similarly, CT and MRI performed on ex vivo tissues have provided insights into TB pathogenesis, including the characterization of excised human TB tissues (106). Multimodality imaging, such as PET/SPECT and CT/MRI, could be combined with other readouts, such as optical imaging and MALDI-MSI, in preclinical models and select clinical scenarios to provide new insights into host-pathogen interactions.

## Implementing molecular imaging: the not-too-distant future

**Figure 2:**
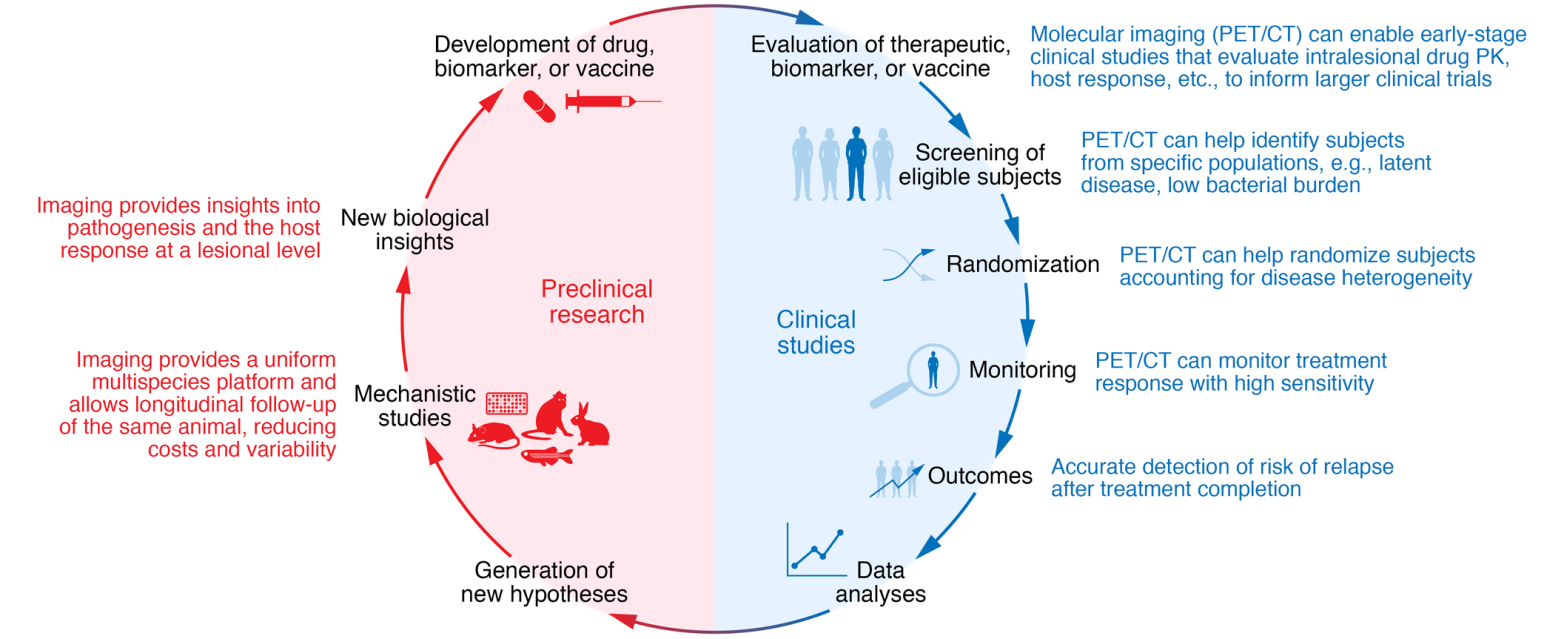
Molecular imaging in the development of new treatments, biomarkers, and vaccines for TB. Molecular imaging can be implemented in the various stages of clinical trials, from early-stage studies to patient screening, randomization, and monitoring of treatment response and outcomes (e.g., relapse), to improve the efficiency and accuracy of TB clinical trials. Additionally, the full spectrum of molecular imaging, including optical imaging and ex vivo techniques, can be employed in preclinical studies. The use of molecular imaging across species allows for crosstalk between preclinical and clinical studies and important collaborative, translational research.

**Figure 3:**
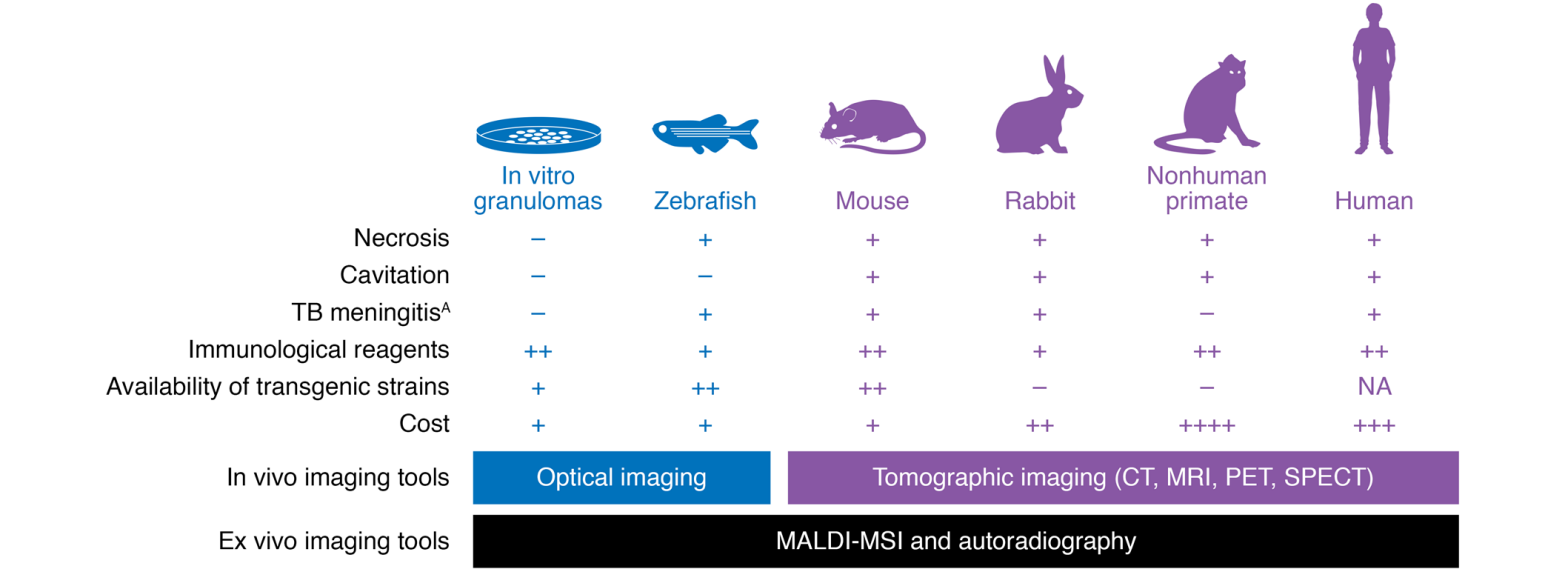
Commonly used TB model systems and their characteristics and utilization in TB imaging research. In vitro granulomas, zebrafish, mice, rabbits, and nonhuman primates are the most common model systems used to recapitulate and study TB. Optical imaging in the zebrafish has been used to study the dynamics of granuloma formation. Mouse and rabbit models have been used to validate multiple imaging tools (PET/SPECT), some of which have been translated into the clinic. ^18^F-FDG PET has also been used extensively in nonhuman primate models. ^A^Although nonhuman primates develop TB meningitis, the characterization of this model has not been reported.

Many molecular imaging techniques can be implemented across multiple animal species as well as for human research (Figure 2). A major advantage of imaging is the ability to follow subjects longitudinally, which can decrease variability and sampling bias, and, for animal studies, substantially decrease the number needed. By decreasing animal numbers, imaging may also improve research costs, which could allow for more efficient experiment. Imaging tools could also be used to stratify disease in preclinical models such that animals assigned to each treatment group have comparable disease. The characteristics of commonly used models for TB and the available imaging tool are summarized in Figure 3. Integration of molecular imaging approaches can complement ex vivo technologies to elucidate mechanisms and to develop novel therapeutics.

Molecular imaging can also benefit clinical research. Although not specific for TB, ^18^F-FDG PET/CT could be used to screen eligible subjects in clinical trials, determine disease heterogeneity to correctly randomize patients between intervention groups, monitor treatment effects, and help predict outcomes (20, 107, 108). However, a standardized methodology to analyze and represent imaging findings should be implemented to allow accurate comparisons between different studies. Artificial intelligence and machine learning may improve data acquisition, reduce scan times, and improve the speed and accuracy of image interpretations (109, 110). Deep learning algorithms have been used to detect features consistent with pulmonary TB in chest radiographs or CT scans, and it is anticipated that computer-aided tools could streamline image analysis as well as improve reproducibility (17, 111). Furthermore, deep learning algorithms could be used to monitor treatment response by detecting obvious and subtle longitudinal changes in imaging biomarkers. Pathogen-specific PET imaging agents, which are currently in development, could serve as specific diagnostic tools with the potential to provide more accurate data on bacterial burden, and thus provide longitudinal information on infection dynamics and treatment responses (39, 40).

Although advanced molecular imaging tools are not uniformly available in all countries, many developing countries are installing and using advanced imaging tools with increased frequency with imaging studies often performed at a substantially lower cost than many developed countries (112). Chest CT can be performed rapidly (seconds) with focused PET scans (3–5 minutes) without the need for sedation, even in young children, and with much lower radiation doses owing to improved CT technologies (4, 113). Additionally, many molecular imaging tracers (PET, SPECT) currently under development for TB are rapidly excreted, which substantially decreases radiation exposure.

It should be noted that although patients with MDR-TB have mortality risks similar to or higher than those of patients with many common cancers (112), the use of radiopharmaceutical imaging is accepted for the management of many cancers (even for children) but is avoided for infectious diseases. The advent of higher-resolution, high-sensitivity PET scanners, such as the EXPLORER total-body PET scanner, could increase the use of PET in both pediatric and adult patients with infectious diseases (114–116). MRI does not expose subjects to ionizing radiation but usually requires longer acquisition times. However, more recently, short-sequence lung MRI has been used for pulmonary imaging in TB patients (117). Additionally, low-field-strength (0.5 T) MRI equipped with state-of-the-art hardware can enable the development of lower-cost MRI machines with good image quality in the lung regions (118). Finally, although specialized equipment (cyclotron) is required to synthesize many PET agents, some radionuclides, such as ^68^Ga, can be synthesized without radionuclide generators and could be produced in remote areas (119). PET agents using ^18^F (e.g., ^18^F-FDG) can also be transported locally, usually within a 2- to 3-hour travel radius. By taking these logistics into consideration, a pragmatic approach could allow for more widespread adoption of imaging technologies for TB.

## Conclusions

In summary, TB is a 21st-century global health threat; however, the field continues to be reliant on several diagnostic and research tools that were developed more than 100 years ago. Next-generation molecular imaging is an emerging technology that affords unparalleled opportunities for visualizing infections, as molecular alterations occur earlier than structural changes. The ability for holistic, 3D spatial characterization and noninvasive, longitudinal monitoring in the same subject is a fundamental advantage over current tools and allows detailed insights into the dynamics and spatiotemporal disease heterogeneity noted with TB. Since many molecular imaging tools are readily available for humans, they could advance fundamental knowledge by enabling basic biology studies in TB patients and accelerate the development of new therapeutics, as well as aid clinical management by serving as precision tools for diagnosis, monitoring, and prognostication.

## Author contributions

AAO, EWT, and SKJ wrote the initial draft and designed the figures. KAL and SM planned and organized the workshop. All authors participated in the workshop as well as writing and editing the report. The order of co–first authors was determined alphabetically.

## Figures and Tables

**Table 3:**
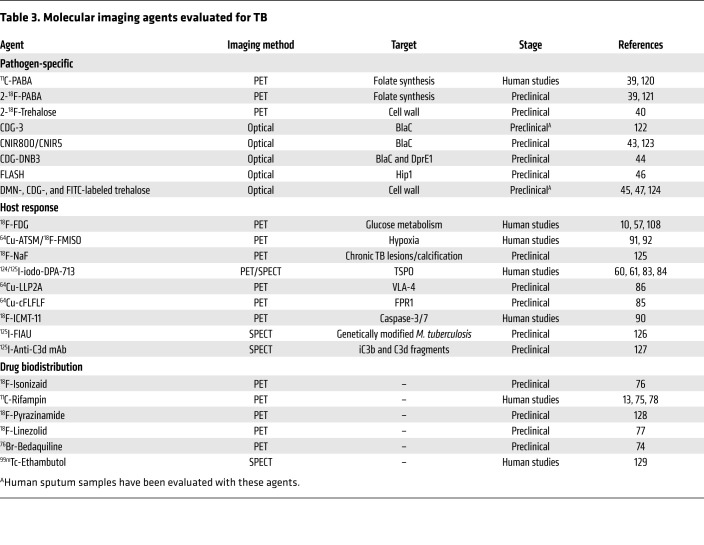
Molecular imaging agents evaluated for TB

**Table 2:**
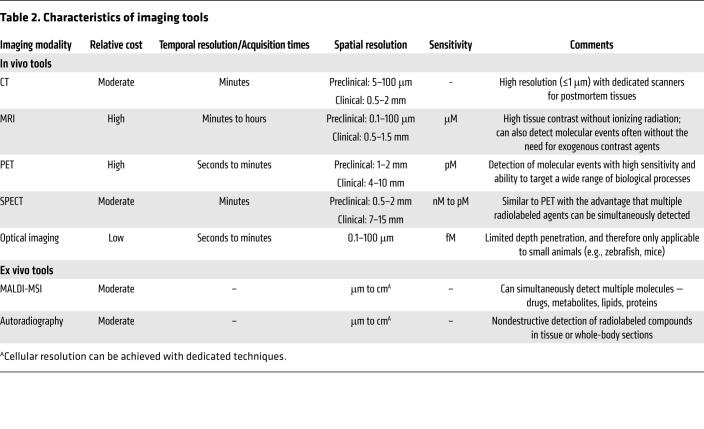
Characteristics of imaging tools

**Table 1:**
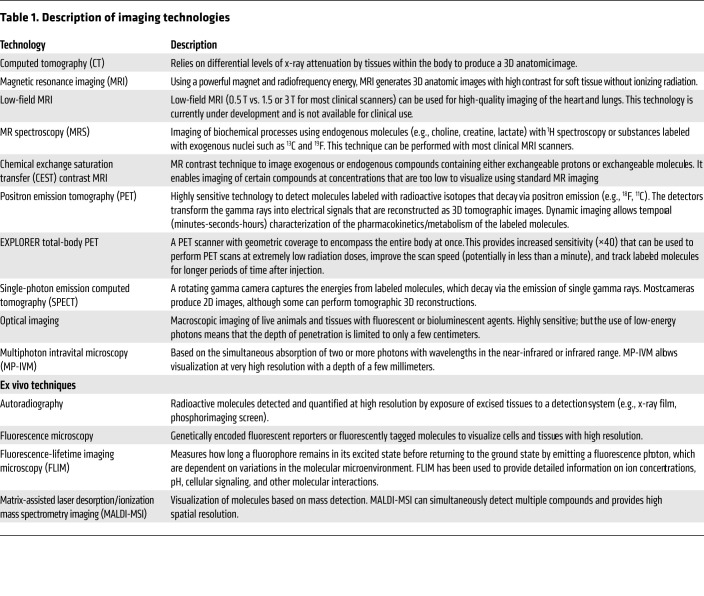
Description of imaging technologies

## Version 1 03/01/2021

Print issue publication
